# Neutrophil responsiveness to IL-10 impairs clearance of *Streptococcus pneumoniae* from the lungs

**DOI:** 10.1093/jleuko/qiad070

**Published:** 2023-06-29

**Authors:** Kadi J Horn, Sam Fulte, Michael Yang, Brian P Lorenz, Sarah E Clark

**Affiliations:** Department of Otolaryngology, University of Colorado School of Medicine, 12700 East 19th Avenue, Aurora, CO 80045, United States; Department of Otolaryngology, University of Colorado School of Medicine, 12700 East 19th Avenue, Aurora, CO 80045, United States; Department of Pathology, University of Colorado School of Medicine, 12631 East 17th Avenue, Aurora, CO 80045, United States; Department of Otolaryngology, University of Colorado School of Medicine, 12700 East 19th Avenue, Aurora, CO 80045, United States; Department of Otolaryngology, University of Colorado School of Medicine, 12700 East 19th Avenue, Aurora, CO 80045, United States

**Keywords:** anti-inflammatory, cytokines, host-pathogen interactions, IL-10, IL-10R, immune evasion, lung infection, myeloid cells, neutrophils, pneumonia, pulmonary, streptococcus pneumoniae

## Abstract

The early immune response to bacterial pneumonia requires a careful balance between pathogen clearance and tissue damage. The anti-inflammatory cytokine interleukin (IL)-10 is critical for restraining otherwise lethal pulmonary inflammation. However, pathogen-induced IL-10 is associated with bacterial persistence in the lungs. In this study, we used mice with myeloid cell specific deletion of IL-10R to investigate the cellular targets of IL-10 immune suppression during infection with *Streptococcus pneumoniae*, the most common bacterial cause of pneumonia. Our findings suggest that IL-10 restricts the neutrophil response to *S. pneumoniae*, as neutrophil recruitment to the lungs was elevated in myeloid IL-10 receptor (IL-10R)–deficient mice and neutrophils in the lungs of these mice were more effective at killing *S. pneumoniae*. Improved killing of *S. pneumoniae* was associated with increased production of reactive oxygen species and serine protease activity in IL-10R–deficient neutrophils. Similarly, IL-10 suppressed the ability of human neutrophils to kill *S. pneumoniae*. Burdens of *S. pneumoniae* were lower in myeloid IL-10R–deficient mice compared with wild-type mice, and adoptive transfer of IL-10R–deficient neutrophils into wild-type mice significantly improved pathogen clearance. Despite the potential for neutrophils to contribute to tissue damage, lung pathology scores were similar between genotypes. This contrasts with total IL-10 deficiency, which is associated with increased immunopathology during *S. pneumoniae* infection. Together, these findings identify neutrophils as a critical target of *S. pneumoniae*-induced immune suppression and highlight myeloid IL-10R abrogation as a mechanism to selectively reduce pathogen burdens without exacerbating pulmonary damage.

## Teaser

We identify neutrophils as a critical cellular target of interleukin-10–directed immune suppression, as neutrophil-mediated clearance of *Streptococcus pneumoniae* is improved in the absence of interleukin-10 receptor.

## Graphical abstract

**Graphical abstract:**
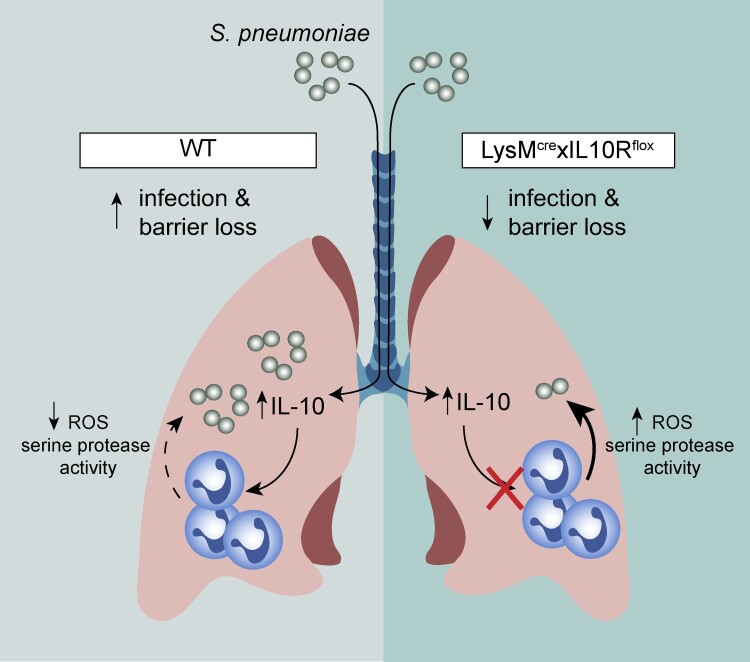

## 1 Introduction

*Streptococcus pneumoniae* (the pneumococcus) is the leading cause of community-acquired pneumonia,^1^ which is particularly deadly in children under 5 years of age and adults over 65 years of age.^2,3^ Pneumococcal lung infections remain common due to the circulation of serotypes not covered by current vaccines and the continued rise of antibiotic resistance.^4,5^ Innate immune cells including macrophages and neutrophils are critical for early clearance of *S. pneumoniae* from the lungs. However, pneumococcal burdens beyond a certain threshold overwhelms innate immune-mediated clearance, and *S. pneumoniae* virulence factors support evasion of phagocytic cell lysis.^6^ The loss of infection control in the lungs is associated with tissue damage and airway barrier dysfunction due to persistent inflammation. The lungs are particularly sensitive to immunopathology, which drives mortality in pneumonia patients even after clearance of the causative agent.^7^ Identifying the key signaling pathways that contribute to pathogen elimination versus those which worsen immunopathology is critical for addressing the clinical burden of pneumococcal pneumonia.

The anti-inflammatory cytokine IL-10 is an important regulator of the immune response to bacterial pneumonia. The IL-10 receptor (IL-10R) comprises IL-10R1 (IL-10 specific) and IL-10R2 (shared with other cytokines) subunits. IL-10 receptor engagement leads to activation of the transcription factor signal transducer activator of transcription 3 (STAT3), which suppresses diverse cellular responses through both transcriptional and posttranscriptional mechanisms.^8,9^ At high infectious doses, IL-10 serves a beneficial role by improving host survival during *S. pneumoniae* infection.^10^ However, in the context of sublethal infection, IL-10 contributes to pathogen persistence.^11^ IL-10 is produced by several immune cell types in the lungs during *S. pneumoniae* infection^10–12^. Macrophage production of IL-10 delays protective IL-17A responses in infant mice infected with *S. pneumoniae.*^13^ Neutrophil-derived IL-10 limits *S. pneumoniae* clearance from the lungs by 48 h postinfection.^10,14^ By 72 to 96 h postinfection, a virulence protein expressed by *S. pneumoniae* called Spr1875 induces IL-10 production in natural killer (NK) cells, which facilitate bacterial persistence in the lung.^11^ NK cell–derived IL-10 limits the expansion of neutrophil, inflammatory monocyte, and alveolar macrophage (AM) populations in the lung.^11^ These observations indicate that lung myeloid cells are affected by the IL-10 response during acute *S. pneumoniae* lung infection.

Neutrophils are the primary producers of alveolar serine proteases including cathepsin G and elastase, which directly contribute to *S. pneumoniae* killing.^15,16^ However, extracellular release of neutrophil elastase can degrade host toll-like receptors and proinflammatory cytokines,^17^ impair macrophage phagocytosis, and degrade the extracellular matrix.^18^ The dual nature of neutrophil activity during pneumococcal infection is exemplified by the effect of neutrophil depletion at different time points. While depletion prior to infection reduces both host survival and *S. pneumoniae* clearance, suggesting a protective role, depletion at 18 h postinfection instead increases host survival.^19^ It is unclear which signaling pathways control the loss of neutrophil-mediated protective activity.

To address the question of how *S. pneumoniae*–induced IL-10 regulates the function of myeloid cells including neutrophils, we generated mice with IL-10R deficiency on LysM^+^ (myeloid) cells. Using this model, we investigated the critical cellular targets of IL-10 mediated suppression of *S. pneumoniae* clearance from the lung. Our goal was to determine whether IL-10 impedes protective antipneumococcal myeloid cell responses. We hypothesized that phagocytic clearance of *S. pneumoniae* would be elevated in the absence of myeloid cell IL-10 responsiveness, resulting in reduced pneumococcal lung burdens but potentially at the cost of increased immunopathology. Our findings indicate that the protective function of neutrophils is improved in the absence of IL-10 signaling, including an increased capacity to kill *S. pneumoniae*. Somewhat unexpectedly, neutrophil-enhanced pathogen clearance did not correlate with worsened tissue pathology, suggesting that IL-10R expression at early time points restrains largely beneficial antibacterial neutrophil activity.

## 2 Materials and methods

### 2.1 Animals

C57BL/6J (wild-type [WT]) and B6.129il10^tm1Cgn^ (*Il10*^−/−^) were purchased from the Jackson Laboratory (stocks #000664 and #002251, respectively). LysM^cre^xIL-10R^flox^ mice were a kind gift from Laurel L. Lenz, University of Colorado. This strain was generated by crossing LysM^cre^ (The Jackson Laboratory; stock #004781) with IL-10R1^flox/flox^ mice.^20^ Cre expression and floxed IL-10R1 were confirmed by polymerease chain reaction. All mice used in experiments were heterozygous for Cre and homozygous for floxed IL-10R1, denoted as LysM^cre^xIL-10R^flox^ mice. Similar results were found between Cre-negative, floxed IL-10R mice (LysM^cre−/−^xIL-10R^flox^; myeloid cell IL-10R maintained) and WT mice. All strains are on the C57BL/6J genetic background. Adult male and female mice were used for these studies at age 6 to 12 wk. Animals were maintained in the University of Colorado Office of Laboratory Animal Resources.

### 2.2 Bacterial infections

A streptomycin-resistant variant of *Streptococcus pneumoniae* serotype 2 strain D39 was used for these studies (kind gift from Dr. Jeffrey N. Weiser, New York University). Bacteria were grown in Todd Hewitt Broth with 0.5% Yeast Extract (BD Bacto) supplemented with 50 μg/mL streptomycin (Sigma) at 37 °C with 5% CO_2_ under static conditions. For infections, *S. pneumoniae* frozen stocks were used to initiate cultures grown to mid-log phase prior to centrifugation at ≥20,000 *g* for 10 min to pellet bacteria, which were then resuspended in phosphate-buffered saline (PBS) at the desired infectious dose. Inoculum burdens were determined by serial dilution for enumeration of colony-forming units (CFUs) injected per mouse. Intratracheal infections conducted in a volume of 50 μL were performed on mice anesthetized with inhaled isoflurane. At the indicated days postinfection, lungs and spleens were homogenized using a Bullet Blender tissue homogenizer (Stellar Scientific) prior to serial dilution on Tryptic Soy agar plates containing neomycin (5 μg/mL; Sigma) and streptomycin (50 μg/mL) prepared with fresh catalase (5,000 units/plate; Worthington Biochemical Corporation). Plates were incubated at 37 °C with 5% CO_2_ overnight (18 h) prior to CFU enumeration. For the neutrophil depletion experiments, mice were injected intraperitoneally 24 h prior to *S. pneumoniae* infection with 200 μg/mouse isotype control IgG2A antibody (Bio X Cell; clone C1.18; catalog #BE0085, lot #722719J2) or anti-Ly6G antibody (Bio X Cell; clone 1A8; catalog #BE0071-1, lot #80772101). Serum cytokines were measured using mouse IL-10, tumor necrosis factor α (TNFα), and interferon γ enzyme-linked immunosorbent assay (ELISA) kits (BD), with analytes detected on a Synergy HT Microplate Reader (BioTek). Cytokines and chemokines collected in 1 mL PBS bronchoalveolar lavage (BAL) fluid were measured using a LEGENDplex panel (catalog #740622; BioLegend), with analytes detected on an LSR Fortessa X-20 in the ImmunoMicro Flow Cytometry Shared Resource Laboratory at the University of Colorado Anschutz Medical Campus (RRID:SCR_021321). BAL MIP-2 (CXCL2) was measured using a mouse CXCL2/MIP-2 ELISA kit (R&D Systems).

### 2.3 Flow cytometry

Flow cytometry was conducted on single cells prepared from lungs of infected mice following transcardial perfusion with 10 mL PBS.^11^ Single cells were prepared by mechanical and enzymatic digestion (DNAseI 30 μg/mL [Sigma] and type 4 collagenase 1 mg/mL (Worthington Biochemical Corporation) followed by filtration (70 μM). Residual red blood cells were lysed using RBC lysis buffer (0.15 M NH_4_Cl, 10 mM KHCO_3_, 0.1 mM Na_2_EDTA, pH 7.4). Cells were incubated in Fc block (anti-CD16/32, 2.4G2 hybridoma supernatant) prior to staining in FACS buffer (1% bovine serum albumin, 0.01% NaN_3_, PBS). Live/dead staining was conducted using a fixable dead cell stain kit (LIVE/DEAD Fixable Blue Dead Cell Stain Kit, #L34961; Thermo Fisher Scientific). For intracellular staining, cells were incubated in Brefeldin A (BD Biosciences) for 4 h prior to staining and permeabilized with 1 mg/mL saponin (Sigma). Reactive oxygen species (ROS) was detected by flow cytometry using the probe DHR-123 (catalog #D1054; Sigma) as previously described.^21^ Briefly, single-cell suspensions were incubated with 10 μg/mL DHR-123 for 20 min at 37 °C prior to washing and staining with cell surface antibodies. All cells were fixed in 1% paraformaldehyde prior to flow cytometric analysis on an LSR Fortessa X-20 in the ImmunoMicro Flow Cytometry Shared Resource Laboratory at the University of Colorado Anschutz Medical Campus (RRID:SCR_021321). Data analysis was performed using FlowJo Software, version 9.9.6 (BD Life Sciences).

The following antibodies were used for this study at a 1:200 dilution: anti-mouse Siglec F (BD; catalog #562681, clone E50-2440, lot #B302914), anti-mouse MHCII (BioLegend; catalog #107643, clone M5/114.15.2, lot #B317262), anti-mouse Ly6G (BioLegend; catalog #127614, clone 1A8, lot #B292772), anti-mouse Ly6C (BioLegend; catalog #128012, clone HK1.4, lot #B250462), anti-mouse CD45.2 (BD; catalog #564616, clone 104, lot #1083734), anti-mouse CD11c (BioLegend; catalog #117338, clone N418, lot #B290360), anti-human/mouse CD11b (BioLegend; catalog #101212, clone M1/70, lot #B281906), anti-mouse TNFα (Thermo Fisher Scientific; catalog #25-7321-82, clone MP6-XT22, lot #2044683), anti-mouse CD210 (BioLegend; catalog #112706, clone 1B1.3A, lot #B282078), anti-human CD45 (BioLegend; catalog #304085, clone HI30, lot #B364861), anti-human CD66b (BioLegend; catalog #392915, clone 6/4 °C, lot #B348382), and anti-human CD14 (BioLegend; catalog #367117, clone 63D3, lot #B262992). In addition to analysis of population percentages and total cell numbers, IL-10R, Ly6G, CD11b, and TNFα expression on murine cells was compared using median fluorescence intensity (MFI).

### 2.4 Lung pathology assessment

Total protein in the BAL fluid was measured using a Pierce BCA protein assay kit (Thermo Fisher Scientific). BAL albumin was measured by ELISA (Eagle Biosciences Inc., Thermo Fisher Scientific) with analytes detected on a Synergy HT Microplate Reader (BioTek). Lung wet/dry ratios were calculated using tissue weight measured postexcision and after incubation at 60 °C for 3 d. For histology, lungs were preserved in 70% EtOH prior to paraffin embedding, tissue slicing, and hematoxylin and eosin staining by the UCD Research Histology Core. High-resolution whole-slide images were collected using Aperio digital pathology slide scanning and analyzed using ImageScope (Leica Biosystems). Histology scores were assigned following blinded analysis (Supplementary Table 1).

### 2.5 Neutrophil functional assays

Bone marrow neutrophils were purified using Histopaque density gradient centrifugation^22^ following isolation from the femurs of naïve mice. Purity was confirmed by flow cytometry (>80% Ly6G^+^ cells). Lung neutrophils were purified from mice 48 h postinfection with *S. pneumoniae* using positive selection with anti-mouse Ly6G-PE antibody (MojoSort PE positive selection kit; BioLegend). Purity was confirmed by flow cytometry (>90% Ly6G^+^ cells). For cell transfer experiments, neutrophils purified from the bone marrow of donor mice were labeled with CFSE (CFSE Cell Division Tracker Kit; BioLegend) prior to transfer of 10^6^ cells per mouse intratracheally into recipient mice anesthetized with inhaled isoflurane. Opsonophagocytic killing assays were conducted with 10^3^ mid-log phase *S. pneumoniae* opsonized with 3% fresh mouse serum (source of complement) for 30 min. During this time, purified neutrophils were incubated with or without recombinant mouse IL-10 for 1 h (100 pg/mL; BioLegend) Following opsonization, bacteria were incubated with 10^5^ neutrophils in Hank’s Balanced Salt Solution with calcium and magnesium (Thermo Fisher Scientific) containing 0.1% glucose (Sigma) for 45 min at 37 °C under rotation. Reactions were stopped by incubation on ice followed by neutrophil lysis using water prior to plating serial dilutions for *S. pneumoniae* CFU enumeration. For killing assays with neutrophils from infected mice, baseline neutrophil CFUs were subtracted from totals. Percent killing was determined relative to control reactions without neutrophils.

Human neutrophils were purified from the blood of healthy adult volunteers who consented to participation under Institutional Review Board approval. Blood was collected into tubes containing acid citrate/dextrose, and neutrophils were isolated by erythrocyte sedimentation followed by Histopaque 1077 centrifugation, as previously described.^23^ Isolated neutrophils were confirmed by flow cytometry to be 90% CD11b ^+^ CD66b ^+^ CD14^−^ cells. Neutrophils were preincubated with or without recombinant human IL-10 (100 ng/mL; BioLegend; catalog #571002) or anti-human IL-10R (1 μg/mL; BioLegend; catalog #308802, clone 3F9) for 30 min at 37 °C prior to killing assays. For killing assays with human neutrophils, 10^5^ neutrophils were incubated with 10^3^*S. pneumoniae*, which was opsonized with baby rabbit serum (10 μL, source of complement) for 30 min at 37 °C in Hank’s Balanced Salt Solution with calcium and magnesium (Thermo Fisher Scientific) containing 0.1% glucose (Sigma) in total volumes of 130 μL. Reactions were incubated at 37 °C for 45 min with rotation. Reactions were stopped on ice followed by plating serial dilutions for *S. pneumoniae* enumeration and calculating percent killing as done previously.

Neutrophil serine protease activity was determined using substrates specific to cathepsin G (0.1 mM Succinyl-Ala-Ala-Pro-Phe-pNA; Sigma) and elastase (0.85 mM MeOSuc-Ala-Ala-Pro-Val-*p*NA; Sigma)^24^ in the presence or absence of 1 × Halt protease inhibitor cocktail (Thermo Fisher Scientific). Neutrophils were incubated with protease inhibitors for 30 min prior to washing, followed by lysis in 0.1% Triton X-100. Substrates were added to cell lysates and incubated for 45 min at 37 °C followed by measurement of absorbance at OD_410_ using a Synergy HT Microplate Reader (BioTek). Substrate activity was calculated relative to control reactions with no neutrophils. Neutrophil ROS was detected using a luminol assay.^25^ Briefly, 10^5^ neutrophils equilibrated for 15 min in KRP buffer (5 mM glucose, 1 mM CaCl_2_, 1 mM MgSO_4_ in PBS) were plated onto Greiner Bio-One LUMITRAC plates (Thermo Fisher Scientific), to which 50 μM luminol (Thermo Fisher Scientific) was added. Luminescence was measured over 1 h at 37 °C using a Synergy HT Microplate Reader (BioTek). The area under the curve was determined relative to control wells with no neutrophils.

### 2.6 Western blotting

Cells were purified by positive selection (MojoSort PE positive selection kit; BioLegend) from the spleens of naïve mice using anti-mouse CD64-PE (BioLegend; clone X54-F/7.1, catalog #139304, lot #B349153) to enrich for myeloid cells and anti-mouse CD3-PE (BioLegend; clone 17A2, catalog #100206, lot #B292677) to enrich for T cells. Cells were exposed to rIL-10 (100 pg/mL) for 1 h prior to lysate preparation. Lysates were run on a 10% sodium dodecyl sulfate–polyacrylamide gel electrophoresis gel under reducing conditions, with protein transferred to nitrocellulose membranes using semi-dry transfer. Membranes were blocked in Odyssey Blocking Buffer (LI-COR) and probed for p-STAT3 (Y705, clone D3A7; Cell Signaling Technology) and β-actin (clone 8H10D10; Cell Signaling Technology). Secondary antibodies against rabbit IgG (LI-COR; anti-rabbit IRDye 800 CW; catalog #827-08365, lot #C90123-1) or mouse IgG (LI-COR; anti-mouse IRDye 680RD; catalog #926-68170, lot #C90715-01) were used to detect expression on an Odyssey imaging system (LI-COR). Images were analyzed using Image Studio software version 5.0.21 (LI-COR).

### 2.7 Study approval

Animal studies were approved by the Animal Care and Use Committee of the University of Colorado School of Medicine (protocol #927). Studies involving biohazardous materials were approved by the Institutional Biosafety Committee (protocol #1418). Human subject studies were approved by the Institutional Review Board (protocol #05-0993).

### 2.8 Statistical analysis

Statistical analyses were conducted using Prism (GraphPad Software; version 9). Data with normal distributions (Shapiro-Wilk test) were analyzed using 2-tailed Student's *t* tests or 1-way analysis of variance for multiple comparisons. Data with non-Gaussian distributions were analyzed using 2-tailed Mann-Whitney *U* tests or Kruskal-Wallis tests for multiple comparisons. *P* values of <0.05 were considered significant.

## 3 Results

### 3.1 IL-10R expression in myeloid cells reduces clearance of *S. pneumoniae*

To generate a mouse strain with defective myeloid cell responsiveness to IL-10R, we crossed LysM^cre^ with IL-10R1^flox/flox^ mice to generate LysM^cre^xIL-10R^flox^ mice, which lack IL-10R1 on LysM^+^ (myeloid lineage) cells.^20,26,27^ To confirm myeloid cell–specific loss of IL-10R function, we compared activation of the transcription factor STAT3 by recombinant IL-10 in CD64^+^ (myeloid cells) vs CD3^+^ (T cells) purified from the spleens of naïve mice. STAT3 activation by rIL-10 was confirmed in T cells from both WT and LysM^cre^xIL-10R^flox^ mice, while activation was lost in myeloid cells from LysM^cre^xIL-10R^flox^ mice (Supplementary Fig. 1A, B). During infection with *S. pneumoniae*, we used flow cytometry to detect IL-10R on the surface of lung myeloid cell populations and found that IL-10R MFI was significantly reduced in myeloid cell types including neutrophils, AMs, dendritic cells (DCs), and inflammatory monocytes in LysM^cre^xIL-10R^flox^ mice compared with WT mice (Supplementary Fig. 1C). In contrast, IL-10R MFI on T cells and NK cells was equivalent between genotypes (Supplementary Fig. 1D).

**Fig. 1.**
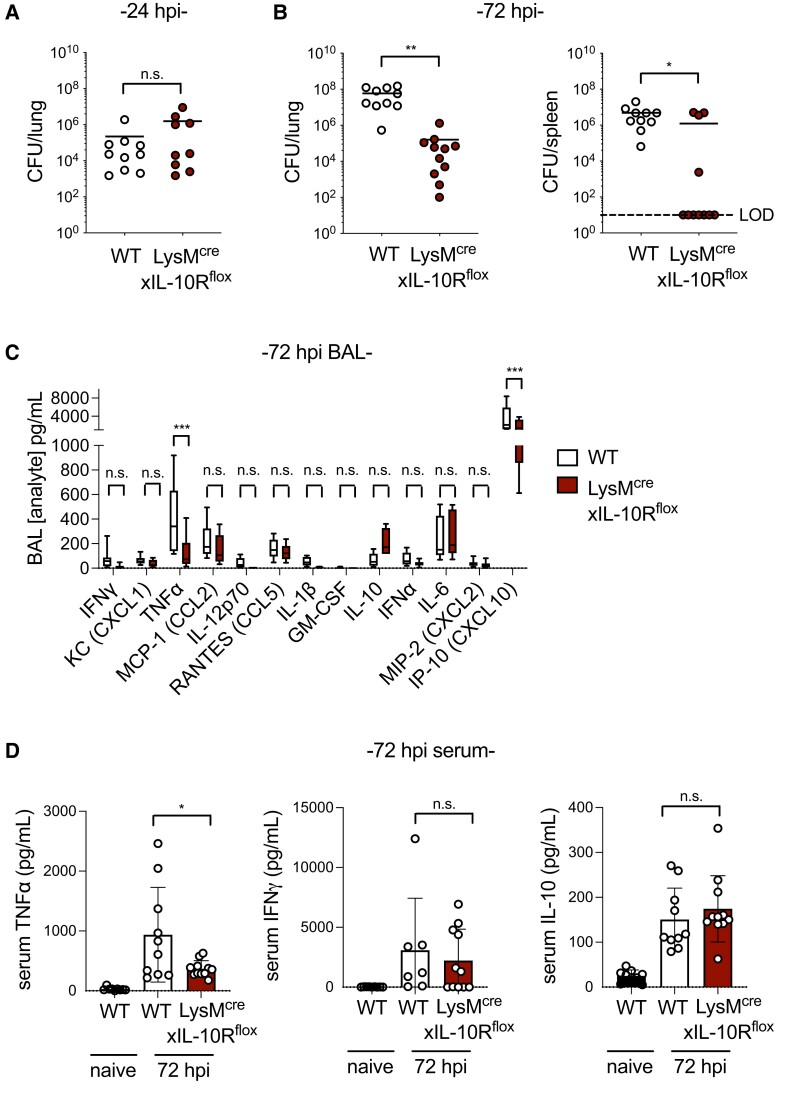
IL-10R expression in myeloid cells reduces clearance of *Streptococcus pneumoniae*. (**A, B**) Lung and spleen *S. pneumoniae* burdens at 24 h (*n* = 9–11 mice/group) (A) and 72 h (*n* = 10–11 mice/group) (B) postinfection with 10^6^ CFU/mouse intratracheally in WT or LysM^cre^xIL-10R^flox^ mice. (**C**) BAL cytokines and chemokines detected in mice 72 h postinfection (*n* = 10–11 mice/group). (**D**) Serum cytokines detected in naïve mice and in mice 72 h postinfection (*n* = 10–11 mice/group). Data are pooled from 3 independent experiments and are displayed as mean ± SEM. *P < 0.05, **P < 0.01, ***P < 0.001; 2-tailed Mann-Whitney *U* test (A, B), 2-way analysis of variance with Sidak's post hoc test (C), or 2-tailed *t* test (D). LOD = limit of detection.

We next evaluated the impact of myeloid IL-10R expression on pneumococcal clearance from the lungs by comparing bacterial burdens in WT and LysM^cre^xIL-10R^flox^ mice. At 24 h postinfection, before a detectable systemic IL-10 response,^11^ there was no difference in lung burdens of *S. pneumoniae* (Fig. 1A). Previously, we found that NK cell–derived IL-10 peaks in the lungs and serum by 72 to 96 h postinfection.^11^ In contrast to 24 h postinfection, at 72 h postinfection bacterial burdens were significantly lower in the lungs of LysM^cre^xIL-10R^flox^ mice compared with WT mice (Fig. 1B). There were also fewer systemic infections in LysM^cre^xIL-10R^flox^ mice, in contrast to WT mice, in which *S. pneumoniae* was detected in the spleens of all mice by 72 h postinfection (Fig. 1B). These data suggest that myeloid cell IL-10R impedes *S. pneumoniae* clearance.

Comparison of the cytokine and chemokine profiles in the BAL fluid of WT and LysM^cre^xIL-10R^flox^ mice at 72 h postinfection revealed that in addition to improved *S. pneumoniae* clearance, LysM^cre^xIL-10R^flox^ mice had significantly lower levels of the proinflammatory cytokine TNFα and the chemokine IP-10 (CXCL10) (Fig. 1C). BAL levels of IL-10 were slightly elevated in LysM^cre^xIL-10R^flox^ mice compared with WT, though this was not statistically significant (Fig. 1C). While 72 h postinfection is past the peak of systemic proinflammatory responses including TNFα and interferon γ, both cytokines were still detected in the serum of infected mice at this time point above the level present in naïve mice, with significantly lower levels of systemic TNFα in LysM^cre^xIL-10R^flox^ mice (Fig. 1D). In contrast, systemic IL-10 was similar between LysM^cre^xIL-10R^flox^ and WT mice (Fig. 1D). As systemic IL-10 is associated with *S. pneumoniae* persistence,^11^ the improved pathogen clearance in LysM^cre^xIL-10R^flox^ mice, despite the availability of systemic and pulmonary IL-10, suggests that IL-10R suppresses myeloid cell–mediated protection against *S. pneumoniae* lung infection.

### 3.2 IL-10R expression limits neutrophil recruitment to the lungs during *S. pneumoniae* infection

**Fig. 2.**
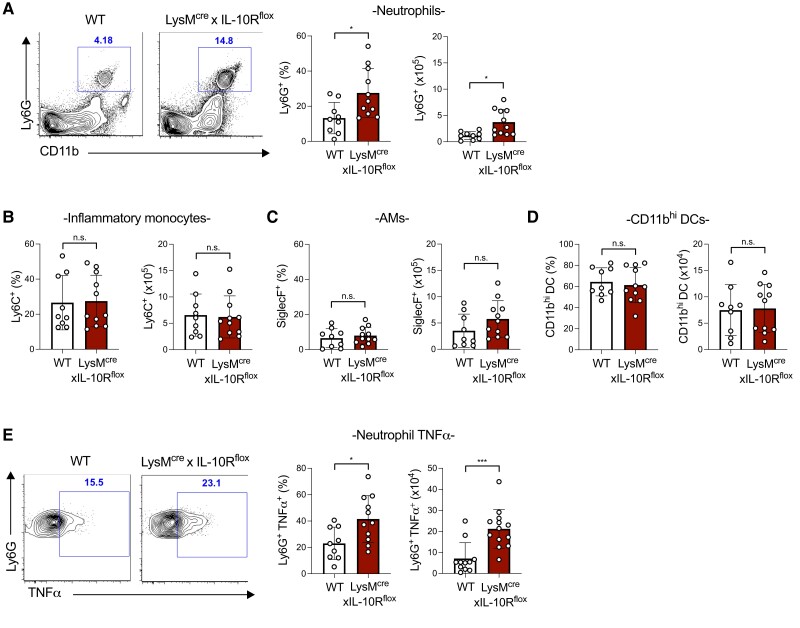
IL-10R expression limits neutrophil recruitment to the lungs during *Streptococcus pneumoniae* infection. (**A–D**) Percentage and total number of neutrophils (CD45 ^+^ SiglecF^−^Ly6G ^+^ CD11b^+^) (A), inflammatory monocytes (CD45 ^+^ SiglecF^−^Ly6G^−^Ly6C ^+^ CD11b^+^) (B), AMs (CD45 ^+^ SiglecF ^+^ CD11b^low^) (C), and CD11b^hi^ DCs (CD45 ^+^ CD11c ^+^ SiglecF^−^MHCII ^+^ CD11b^hi^) (D) detected by flow cytometry in the lungs at 72 h postinfection with *S. pneumoniae* 10^6^ CFU/mouse intratracheally in WT or LysM^cre^xIL-10R^flox^ mice (*n* = 10–11 mice/group). (**E**) Percentage and total number of TNFα^+^ neutrophils from population gated in panel A detected by intracellular flow cytometry. Representative plots are shown for neutrophil and neutrophil TNFα gates. Data are pooled from 3 independent experiments and are displayed as mean ± SEM. *P < 0.05, ***P < 0.001 2-tailed *t* test.

We next used flow cytometry to determine the impact of IL-10R on individual myeloid cell populations in the lungs following *S. pneumoniae* infection. Previously, we found that in *Il10*^−/−^ mice, there was an increased infiltration of several myeloid cell populations to the lungs, including inflammatory monocytes and neutrophils, as well as an elevated number of alveolar macrophages.^11^ In contrast, we observed a selective impact on neutrophil recruitment in LysM^cre^xIL-10R^flox^ mice. By 72 h postinfection, there were significantly increased numbers of neutrophils in the lungs of myeloid cell IL-10R–deficient mice compared with WT mice (Fig. 2A). In contrast, populations of inflammatory monocytes, AMs, and inflammatory CD11b^hi^ DCs remained unchanged between genotypes (Fig. 2B–D). Differential neutrophil recruitment resulted in a higher percentage and total number of neutrophils producing TNFα in the lungs of LysM^cre^xIL-10R^flox^ mice compared with WT mice (Fig. 2E). In contrast, there was no detectable difference in TNFα production by other lung myeloid cell types including inflammatory monocytes, AMs, and CD11b^hi^ DCs (Supplementary Fig. 2). These findings suggest that myeloid cell expression of IL-10R suppresses neutrophil recruitment during *S. pneumoniae* lung infection.

Lung neutrophils are a heterogeneous population, with varying developmental and activation states during pneumococcal infection. Compared with neutrophils in the lungs of WT mice at 72 h postinfection, neutrophils from LysM^cre^xIL-10R^flox^ mice had elevated Ly6G MFI, which has been associated with improved pneumococcal clearance in the spleen,^16^ while CD11b and TNFα MFIs were comparable between genotypes (Supplementary Fig. 3A). In addition to TNFα, ∼38% of lung neutrophils in WT mice expressed ROS at 72 h postinfection (Supplementary Fig. 3B), indicative of activation. However, IL-10R expression increased during pneumococcal infection in WT mice, as the percentage of neutrophils with detectable surface IL-10R expression was significantly elevated by 72 h postinfection compared with naïve mice (Supplementary Fig. 3C). This finding suggests that neutrophil activation at 72 h postinfection may be counterbalanced by the expression of IL-10R.

### 3.3 Neutrophil killing of *S. pneumoniae* is enhanced in the absence of IL-10R

**Fig. 3.**
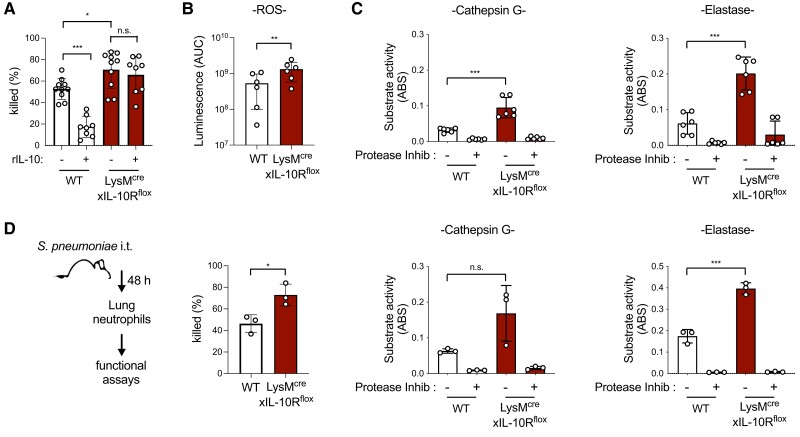
Neutrophil killing of *Streptococcus pneumoniae* is enhanced in myeloid IL-10R–deficient mice. (**A**) Percent of *S. pneumoniae* killed by neutrophils purified from the bone marrow of naïve WT or LysM^cre^xIL-10R^flox^ mice ± preincubation rIL-10 (*n* = cells isolated from 8–10 mice/group). (**B**) Total ROS measured by luminol luminescence (area under the curve [AUC]) produced in 1 h by neutrophils purified from the bone marrow of WT or LysM^cre^xIL-10R^flox^ mice (cells isolated from 6 mice/group). (**C**) Serine protease activity for cathepsin G and elastase ± protease inhibitor cocktail detected by substrate cleavage for neutrophils purified from the bone marrow of naïve WT or LysM^cre^xIL-10R^flox^ mice (cells isolated from 6 mice/group). (**D**) Percent of *S. pneumoniae* killed and serine protease activity for neutrophils purified from the lungs of WT or LysM^cre^xIL-10R^flox^ mice 48 h postinfection with *S. pneumoniae* 10^6^ CFU/mouse intratracheally (i.t.) ( cells isolated from 3 mice/group), schematic shown on left. Data are pooled from 3 independent experiments (A–C) or representative from 1 of 3 independent experiments (D), displayed as mean ± SEM. *P < 0.05, **P < 0.01, ***P < 0.001; 1-way analysis of variance with Tukey's post hoc test (A) or 2-tailed *t* test (B–D). ABS = absorbance.

The enhanced neutrophil recruitment we observed in LysM^cre^xIL-10R^flox^ mice infected with *S. pneumoniae* prompted our investigation of the impact of IL-10R expression on neutrophil-mediated killing of *S. pneumoniae*. Neutrophils isolated from the bone marrow of naïve LysM^cre^xIL-10R^flox^ mice had a slightly enhanced capacity to kill *S. pneumoniae* compared with neutrophils from naïve WT mice (Fig. 3A). The addition of rIL-10 suppressed killing of *S. pneumoniae* by neutrophils from WT mice, as others have shown.^28^ In contrast, rIL-10 had no impact on the ability of neutrophils from LysM^cre^xIL-10R^flox^ mice to kill *S. pneumoniae* (Fig. 3A). Unlike neutrophils, AMs from naïve LysM^cre^xIL-10R^flox^ mice had a reduced capacity to kill *S. pneumoniae* compared with AMs from WT mice (Supplementary Fig. 4A), indicating selective restriction of neutrophil killing by IL-10R signaling. The two primary mechanisms of neutrophil-mediated killing of *S. pneumoniae* involve the production of ROS and the activity of serine proteases including cathepsin G and elastase. Consistent with the enhanced baseline killing of neutrophils in LysM^cre^xIL-10R^flox^ mice, we found that neutrophils purified from the bone marrow of these mice had elevated production of ROS (Fig. 3B, Supplementary Fig. 4B) as well as cathepsin G and elastase activity (Fig. 3C). To determine whether these phenotypes translated to increased neutrophil functional activity in the lungs during *S. pneumoniae* infection, we purified neutrophils from the lungs of WT and LysM^cre^xIL-10R^flox^ mice at 48 h postinfection. Lung neutrophils from LysM^cre^xIL-10R^flox^ mice had improved killing of *S. pneumoniae* and higher levels of elastase activity compared with lung neutrophils from WT mice (Fig. 3D). These findings indicate that IL-10R expression restricts neutrophil functional activity, including killing of *S. pneumoniae*.

**Fig. 4.**
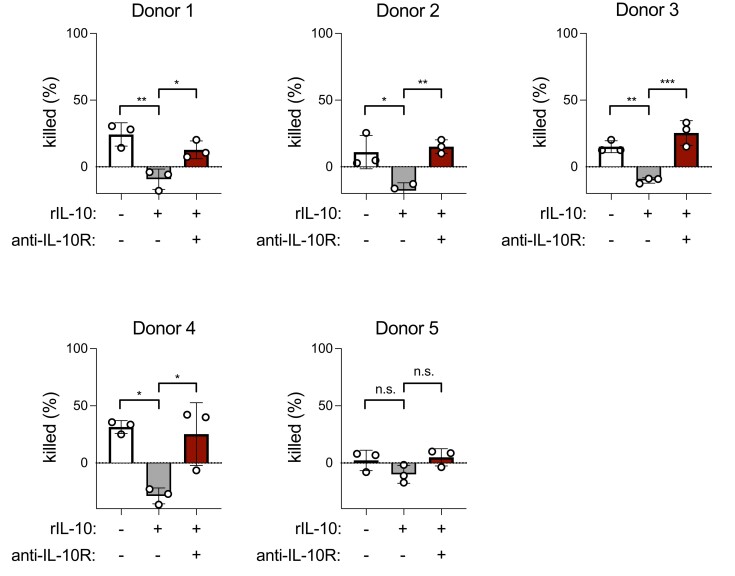
IL-10 reduces killing of *Streptococcus pneumoniae* by human neutrophils. Percent of *S. pneumoniae* killed by neutrophils purified from the peripheral blood of healthy human donors ± preincubation with rIL-10 or anti-IL-10R antibody (*n* = 5 donors, killing assays from each donor run in triplicate). *P < 0.05, **P < 0.01, ***P < 0.001; 1-way analysis of variance with Tukey's post hoc test.

To determine whether the impact of IL-10 on *S. pneumoniae* killing translated to human neutrophils, we compared killing of *S. pneumoniae* by neutrophils isolated from healthy human donors with or without the addition of recombinant IL-10. Additional reactions included rIL-10 together with anti-human IL-10R antibody to block IL-10 signaling. For all donors in which neutrophil killing was observed, the addition of rIL-10 significantly reduced *S. pneumoniae* killing, and this effect was abrogated by the inclusion of anti-IL-10R antibody (Fig. 4). Together, these data suggest that IL-10R signaling suppresses both murine and human neutrophil killing of *S. pneumoniae*.

### 3.4 *S. pneumoniae* clearance is improved by IL-10R–deficient neutrophils

**Fig. 5.**
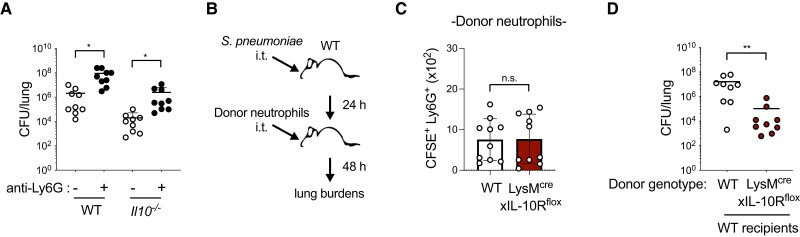
*Streptococcus pneumoniae* clearance is improved by IL-10R–deficient neutrophils. (**A**) Lung burden of *S. pneumoniae* detected at 72 h postinfection with 10^6^ CFU/mouse intratracheally (i.t.) in WT or *Il10*^−/−^ mice treated with isotype control (−) or anti-Ly6G antibodies (+) intraperitoneally 24 h prior to *S. pneumoniae* infection (*n* = 9 mice/group). (**B**) Schematic of neutrophil adoptive transfer. (**C, D**) Donor neutrophils labeled with CFSE detected by flow cytometry (C) and lung burden of *S. pneumoniae* (D) in recipient WT mice 72 h postinfection with *S. pneumoniae* 10^6^ CFU/mouse i.t., with adoptive transfer of neutrophils purified from WT or LysM^cre^xIL-10R^flox^ mice at 24 h postinfection (*n* = 9 recipient mice/group). Data are pooled from 3 independent experiments and are displayed as mean ± SEM. **P < 0.01; Kruskal-Wallis with Dunn's post hoc test (A) or Mann-Whitney *U* test (C-D).

We next compared the impact of neutrophil depletion on *S. pneumoniae* infection in WT and *Il10*^−/−^ mice using anti-Ly6G antibody treatment (Supplementary Fig. 4C, D). Neutrophil depletion prior to infection reduced clearance of *S. pneumoniae* in WT mice (Fig. 5A), suggesting a protective role at early time points, as others have shown.^19^ In *Il10*^−/−^ mice, neutrophil depletion had a similar impact, resulting in increased *S. pneumoniae* burdens compared with nondepleted mice (Fig. 5A). These findings indicate the capacity for neutrophil-mediated protection in the absence of IL-10. To test the hypothesis that neutrophils which are no longer responsive to IL-10 improve clearance of *S. pneumoniae*, we conducted an adoptive transfer experiment in which neutrophils isolated from the bone marrow of naïve WT or LysM^cre^xIL-10R^flox^ mice were injected into the lungs of WT mice at 24 h postinfection (Fig. 5B). Neutrophils purified from either genotype had equivalent survival ex vivo as determined by live/dead staining (Supplementary Fig. 4E), suggesting similar baseline viability. Four hours following transfer, donor neutrophils labeled with CFSE comprised a significant portion of total neutrophils in the BAL (∼30%) and a minor population in lung tissue (∼7%) of recipient mice, which declined over time (Supplementary Fig. 4F). By 72 h postinfection, the small number of donor neutrophils remaining was similar between genotypes (Fig. 5C). In contrast to recipients of WT neutrophils, recipients of LysM^cre^xIL-10R^flox^ neutrophils had reduced lung burdens of *S. pneumoniae* (Fig. 5D). The reduced pathogen burdens in recipients of LysM^cre^xIL-10R^flox^ neutrophils suggests that IL-10 responsiveness impairs neutrophil-mediated clearance of *S. pneumoniae* from the lungs.

### 3.5 Limited impact of myeloid cell IL-10R expression on lung immunopathology during acute infection

**Fig. 6.**
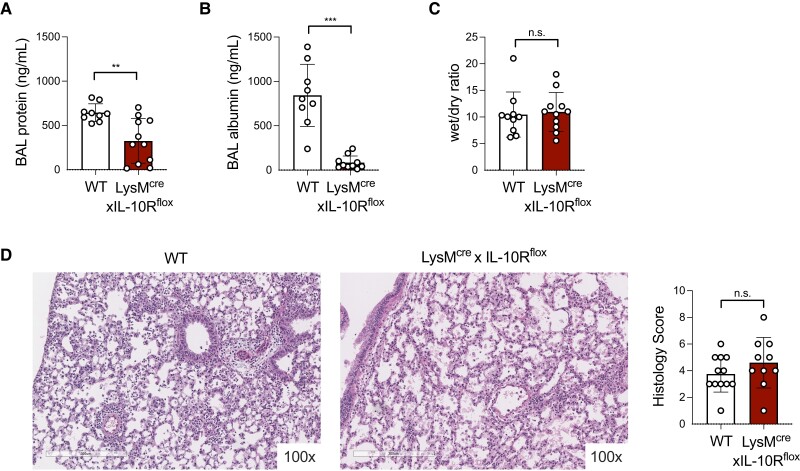
Limited impact of myeloid cell IL-10R expression on lung immunopathology during acute infection. (**A, B**) Total protein (A) and albumin (B) detected in bronchoalveolar lavage fluid collected from WT or LysM^cre^xIL-10R^flox^ mice 72 h postinfection with *Streptococcus pneumoniae* 10^6^ CFU/mouse intratracheally (*n* = 9–11 mice/group). (**C**) Wet/dry ratio detected for lungs collected 72 h postinfection from WT or LysM^cre^xIL-10R^flox^ mice (*n* = 10–11 mice/group). (**D**) Representative lung tissue sections stained with hematoxylin and eosin at ×100 magnification and histopathological scores for lung tissue collected 72 h postinfection from WT or LysM^cre^xIL-10R^flox^ mice (*n* = 10–12 mice/group). Data are pooled from 3 independent experiments and are displayed as mean ± SEM. **P < 0.01, ***P < 0.001; 2-tailed *t* test.

Neutrophils contribute to early *S. pneumoniae* killing but can also induce lung damage. To establish the impact of myeloid IL-10R expression on lung immunopathology, we compared measurements of airway barrier permeability and tissue damage in the lungs of WT and LysM^cre^xIL-10R^flox^ mice at 72 h postinfection. We found that LysM^cre^xIL-10R^flox^ mice had reduced total protein and albumin detected in the BAL compared with WT mice (Fig. 6A-B), correlating with improved *S. pneumoniae* clearance at this time point. Using the lung wet-to-dry ratio as an indicator of pulmonary edema, we found no difference between WT and LysM^cre^xIL-10R^flox^ infected mice (Fig. 6C). Lung histopathological scores were also similar between genotypes (Fig. 6D, Supplementary Table 1). These findings suggest that myeloid cell IL-10R deficiency does not exacerbate lung immunopathology in mice infected with *S. pneumoniae*.

## 4 Discussion

Our findings identify neutrophils as a key cellular target of *S. pneumoniae*–induced immune suppression during acute pulmonary infection. In the absence of myeloid IL-10R expression, neutrophils had an enhanced capacity to kill *S. pneumoniae*, which was associated with improved pulmonary clearance. These observations were confirmed in human neutrophils, as IL-10 signaling reduced *S. pneumoniae* killing. In prior work, we found that *S. pneumoniae* induces a systemic NK cell–dependent IL-10 response by 72 h postinfection.^11^ In that study, transfer of WT NK cells into the lungs of IL-10–deficient recipient mice reduced neutrophil recruitment.^11^ Here, we build on these findings by demonstrating that myeloid cell IL-10R is responsible for the impaired neutrophil recruitment induced by IL-10 during *S. pneumoniae* infection. Together, these data implicate *S. pneumoniae*-induced IL-10 in the restraint of neutrophil-mediated protection against pneumococcal infection.

Neutrophils themselves are a source of IL-10 prior to peak induction of the systemic NK cell IL-10 response. IL-10 production in neutrophils is limited by expression of the ectonucleotidase CD73, which generates extracellular adenosine, and by the transcription factor Krüppel-like factor 4 (KLF4).^28,29^ Regulation of neutrophil-mediated killing by adenosine signaling, including CD73 and the adenosine receptor A2B, primarily impacts neutrophil production of ROS,^28,30^ in contrast to the broader effect that we observed on both ROS and serine protease activity for IL-10R–deficient neutrophils. The reduced neutrophil killing reported in the absence of KLF4 and CD73, when neutrophils are secreting more IL-10, may therefore be amplified by IL-10R feedback. IL-10 has distinct cell type–specific effects on gene transcription.^31^ In neutrophils, IL-10 signaling through the JAK/STAT3 pathway reduces the transcription of cytokines including TNFα and suppresses ROS production through the inhibition of extracellular signal regulated kinase 1/2 activity.^32,33^ The connection between IL-10 signaling and serine protease activity remains uncharacterized and is likely indirect, as serine proteases including cathepsin G and elastase are stored intracellularly in azurophilic granules, with release regulated by several proteins including clade A and B serpins.^34^ Together, these findings imply that neutrophil killing is regulated by both neutrophil-derived IL-10 and neutrophil expression of IL-10R.

Neutrophils which are nonresponsive to IL-10 are sufficient to improve *S. pneumoniae* clearance from the lungs, as suggested by the reduced pneumococcal lung burdens we observed in recipients of IL-10R–deficient neutrophils. These findings complement a recent study demonstrating that the reverse is also true; transfer of WT neutrophils which can produce IL-10, and are less functional, reduce *S. pneumoniae* clearance from the lungs of IL-10–deficient recipients.^14^ In either case, *S. pneumoniae* clearance is affected by neutrophil phenotype, with neutrophil-associated IL-10 production and signaling resulting in defective clearance. In human neutrophils, IL-10R expression is increased in response to inflammatory stimuli,^35^ as with our observation of increased surface IL-10R expression during pneumococcal infection. Neutrophils from septic patients have elevated IL-10R expression, and neutrophil production of proinflammatory cytokines is reduced following IL-10 exposure.^33^ Similarly, we found that IL-10 limited the ability of human neutrophils to kill *S. pneumoniae*, consistent with prior studies demonstrating that IL-10 reduces human neutrophil phagocytosis and killing of other bacteria.^36,37^ These data indicate that IL-10 signaling limits neutrophil-mediated killing of *S. pneumoniae*.

IL-10 regulates lung immunopathology during bacterial pneumonia, as tissue damage is elevated in IL-10–deficient mice.^10^ Strikingly, the heightened neutrophil functional activity that we observed in the absence of myeloid IL-10R did not come at the cost of worsened immunopathology by 72 h postinfection. While the number of TNFα^+^ neutrophils in myeloid IL-10R–deficient mice was elevated, total pulmonary proinflammatory cytokine and chemokine production was either equivalent to WT, or in the case of TNFα and IP-10, levels were reduced. Histology scores were similar between genotypes, and lower BAL albumin and protein content suggest reduced alveolar-capillary membrane permeability in myeloid IL-10R–deficient mice. It is unclear why the lung wet/dry ratios, serving as an indicator of pulmonary edema, were not also reduced in myeloid IL-10R–deficient mice, though this is considered a less precise measurement compared with BAL albumin.^38^ Regardless, we did not find evidence for increased lung immunopathology in the myeloid IL-10R–deficient mice, despite the loss of IL-10 signaling in myeloid cells.

Regarding the absence of a barrier defect in myeloid IL-10R–deficient mice, one possibility is that other lung cell types are sufficient to maintain barrier integrity. The lack of IL-10R expression in epithelial cells^39^ indicates lymphocytes such as tissue resident innate lymphoid cells or T cells as probable targets. Neutrophils produce several factors which are cytotoxic, including ROS, proteases, and proinflammatory cytokines, which can induce cell death indirectly.^40^ Among these, NADPH oxidase-dependent ROS production was not responsible for increased airway barrier permeability induced by neutrophil influx,^41^ though the role of mitochondrial ROS was not evaluated. In the context of acute respiratory distress syndrome, immunopathology is associated with increased lung neutrophils.^42^ However, neutrophils in the lungs of acute respiratory distress syndrome patients are dysfunctional, with a reduced capacity to kill pathogens and increased release of neutrophil extracellular traps, which contain several proteases.^42,43^ Extracellular release of neutrophil elastase, which occurs during NETosis, damages the extracellular matrix, contributing to tissue pathology.^44,45^ Together, these studies suggest that neutrophil-mediated damage is elevated in the context of neutrophil dysfunction and the release of extracellular proteases. The elevated damage coincident with neutrophil dysfunction may differ from a setting where neutrophil functional activity is increased, as we observe in the absence of myeloid IL-10R. For example, myeloid-targeted deletion of KLF4 increased *S. pneumoniae* burdens, lung pathology, and barrier permeability, despite elevated systemic IL-10.^46^ Together with our findings of increased neutrophil-mediated killing, bacterial clearance, and barrier function in myeloid IL-10R–deficient mice, this suggests that total pathogen burden is a more informative indicator of pulmonary tissue damage during pneumococcal pneumonia than the presence of neutrophils alone*. S. pneumoniae* also contributes to tissue damage through expression of the pore-forming toxin pneumolysin (ply). Ply is cytotoxic and increases release of extracellular neutrophil elastase.^18^ Pneumococcal-driven immunopathology and neutrophil dysfunction could therefore serve as early indicators of airway barrier loss, while neutrophils with reduced IL-10 responsiveness may instead have a largely beneficial role during acute lung infection.

Beyond *S. pneumoniae*, diverse bacteria activate production of IL-10, including *Mycobacterium bovis* BCG and *Bordatella pertussis.*^47,48^ Clearance of both *M. bovis* BCG and *B. pertussis* is improved in the absence of IL-10, and neutrophil depletion results in increased BCG burdens,^47^ as with *S. pneumoniae*. *Bordatella*-induced IL-10 suppresses neutrophil infiltration,^48^ as we find during pneumococcal infection. There are numerous cellular targets of IL-10 during bacterial infection. IL-10 induced by *Staphylococcus aureus* suppresses Th17 and IL-22 responses, leading to reduced bacterial clearance^49^ and increased systemic infection.^50^ Blocking IL-10R improves control of *Mycobacterium tuberculosis* infection as well as BCG-induced memory T cell responses.^51^ It is unclear whether loss of IL-10R similarly impacts the induction of vaccine immunity against *S. pneumoniae*. As with the positive feedback indicated for neutrophils, a toxin produced by group B *Streptococcus* induces macrophage IL-10, which suppresses macrophage activation.^52^ These studies highlight IL-10–mediated suppression of otherwise protective immune activity as a recurring theme during microbial infection. Our data indicate that neutrophils are a key cellular target of the IL-10 response induced by *S. pneumoniae* during acute lung infection.

In addition to myeloid cells, alveolar type 2 cells express LysM during development.^53^ However, the lack of IL-10R expression in epithelial cells including alveolar type 2 cells^54^ limits the likelihood of an impact in the setting of our LysM^cre^xIL-10R^flox^ model. For this study, we restricted our analysis to early time points following pneumococcal infection. Kinetic analysis of responses beyond 72 h postinfection will be important in future studies to determine whether the abrogation of neutrophil responsiveness to IL-10 has a longer-term impact on recovery from pneumococcal infection. In addition, restraint of neutrophil activity may be critical for the defense against *S. pneumoniae* in other contexts, such as during bacterial or viral co-infections as well as following invasion to other host sites.

In summary, we find that loss of IL-10 responsiveness in myeloid cells has a positive impact on neutrophil-mediated clearance of *S. pneumoniae* from the lungs during acute infection. These findings denote a rare point of separation between innate immune responses that contribute to pathogen clearance versus those that exacerbate barrier damage, as the loss of IL-10R expression in myeloid cells improved *S. pneumoniae* killing without associated tissue damage during acute lung infection. Targeting IL-10R signaling on myeloid cells including neutrophils may therefore improve the innate immune response to pneumococcal pneumonia.

## Supplementary Material

qiad070_Supplementary_Data

## References

[1] Torres A , Cilloniz C, Niederman MS, Menéndez R, Chalmers JD, Wunderink RG, van der Poll T. Pneumonia. Nat Rev Dis Primers. 2021:7(1):25. 10.1038/s41572-021-00259-0 33833230

[2] Naucler P , Darenberg J, Morfeldt E, Örtqvist Å, Normark BH. Contribution of host, bacterial factors and antibiotic treatment to mortality in adult patients with bacteraemic pneumococcal pneumonia. Thorax. 2013:68(6):571–579. 10.1136/thoraxjnl-2012-203106 23442364

[3] Marangu D , Zar HJ. Childhood pneumonia in low-and-middle-income countries: an update. Paediatr Respir Rev. 2019:32:3–9. 10.1016/j.prrv.2019.06.001 31422032 PMC6990397

[4] Cherazard R , Epstein M, Doan T-L, Salim T, Bharti S, Smith MA. Antimicrobial resistant Streptococcus pneumoniae: prevalence, mechanisms, and clinical implications. Am J Ther. 2017:24(3):e361–e369. 10.1097/MJT.0000000000000551 28430673

[5] Balsells E , Guillot L, Nair H, Kyaw MH. Serotype distribution of Streptococcus pneumoniae causing invasive disease in children in the post-PCV era: a systematic review and meta-analysis. PLoS One. 2017:12(5):e0177113-20. 10.1371/journal.pone.0177113 28486544 PMC5423631

[6] Weiser JN , Ferreira DM, Paton JC. Streptococcus pneumoniae: transmission, colonization and invasion. Nat Rev Microbiol. 2018:16(6):355–367. 10.1038/s41579-018-0001-8 29599457 PMC5949087

[7] Quinton LJ , Walkey AJ, Mizgerd JP. Integrative physiology of pneumonia. Physiol Rev. 2018:98(3):1417–1464. 10.1152/physrev.00032.2017 29767563 PMC6088146

[8] Rossato M , Cencig S, Gasperini S, Cassatella MA, Bazzoni F. IL-10 modulates cytokine gene transcription by protein synthesis-independent and dependent mechanisms in lipopolysaccharide-treated neutrophils. Eur J Immunol. 2007:37(11):3176–3189. 10.1002/eji.200737625 17948269

[9] Murray PJ . Understanding and exploiting the endogenous interleukin-10/STAT3-mediated anti-inflammatory response. Curr Opin Pharmacol. 2006:6(4):379–386. 10.1016/j.coph.2006.01.010 16713356

[10] Peñaloza HF , Nieto PA, Mũnoz-Durango N, Salazar-Echegarai FJ, Torres J, Parga MJ, Alvarez-Lobos M, Riedel CA, Kalergis AM, Bueno SM. Interleukin-10 plays a key role in the modulation of neutrophils recruitment and lung inflammation during infection by Streptococcus pneumoniae. Immunology. 2015:146(1):100–112. 10.1111/imm.12486 26032199 PMC4552505

[11] Clark SE , Schmidt RL, Aguilera ER, Lenz LL. IL-10-producing NK cells exacerbate sublethal Streptococcus pneumoniae infection in the lung. Transl Res. 2020:226:70–82. 10.1016/j.trsl.2020.07.001 32634590 PMC7572800

[12] van der Poll T , Marchant A, Keogh CV, Goldman M, Lowry SF. Interieukin-10 impairs host defense in murine pneumococcal pneumonia. J Infect Dis. 1996:174(5):994–1000. 10.1093/infdis/174.5.994 8896500

[13] Bogaert D , Weinberger D, Thompson C, Lipsitch M, Malley R. Impaired innate and adaptive immunity to Streptococcus pneumoniae and its effect on colonization in an infant mouse model. Infect Immun. 2009:77(4):1613–1622. 10.1128/iai.00871-08 19168741 PMC2663178

[14] González LA , Melo-González F, Sebastián VP, Vallejos OP, Noguera LP, Suazo ID, Schultz BM, Manosalva AH, Peñaloza HF, Soto JA, et al Characterization of the anti-inflammatory capacity of IL-10-producing neutrophils in response to Streptococcus pneumoniae infection. Front Immunol. 2021:12:638917. 10.3389/fimmu.2021.638917 33995357 PMC8113954

[15] Hahn I , Janze A-K, Steinwede K, Ding N, Bohling J, Brumshagen C, Serrano H, Gauthier F, Paton JC, Welte T, et al Cathepsin G and neutrophil elastase play critical and nonredundant roles in lung-protective immunity against Streptococcus pneumoniae in mice. Infect Immun. 2011:79(12):4893–4901. 10.1128/IAI.05593-11 21911460 PMC3232647

[16] Deniset JF , Surewaard BG, Lee W-Y, Kubes P. Splenic Ly6Ghigh mature and Ly6Gint immature neutrophils contribute to eradication of S. pneumoniae. J Exp Med. 2017:214(5):1333–1350. 10.1084/jem.20161621 28424248 PMC5413339

[17] Domon H , Nagai K, Maekawa T, Oda M, Yonezawa D, Takeda W, Hiyoshi T, Tamura H, Yamaguchi M, Kawabata S, et al Neutrophil elastase subverts the immune response by cleaving toll-like receptors and cytokines in pneumococcal pneumonia. Front Immunol. 2018:9:732. 10.3389/fimmu.2018.00732 29922273 PMC5996908

[18] Domon H , Oda M, Maekawa T, Nagai K, Takeda W, Terao Y. Streptococcus pneumoniae disrupts pulmonary immune defence via elastase release following pneumolysin-dependent neutrophil lysis. Sci Rep. 2016:6(1):38013. 10.1038/srep38013 27892542 PMC5125098

[19] Ghanem ENB , Clark S, Roggensack SE, McIver SR, Alcaide P, Haydon PG, Leong JM. Extracellular adenosine protects against Streptococcus pneumoniae lung infection by regulating pulmonary neutrophil recruitment. PLoS Pathog. 2015:11(8):e1005126. 10.1371/journal.ppat.1005126 26313746 PMC4552087

[20] Pils MC , Pisano F, Fasnacht N, Heinrich J-M, Groebe L, Schippers A, Rozell B, Jack RS, Müller W. Monocytes/macrophages and/or neutrophils are the target of IL-10 in the LPS endotoxemia model. Eur J Immunol. 2010:40(2):443–448. 10.1002/eji.200939592 19941312

[21] Pioch J , Blomgran R. Optimized flow cytometry protocol for dihydrorhodamine 123-based detection of reactive oxygen species in leukocyte subpopulations in whole blood. J Immunol Methods. 2022:507:113308. 10.1016/j.jim.2022.113308 35760097

[22] Swamydas M , Luo Y, Dorf ME, Lionakis MS. Isolation of mouse neutrophils. Curr Protoc Immunol. 2015:110(1):7.23.1–21. 10.1002/0471142735.im0320s110 PMC4574512 26237011

[23] Antonio M-B , Arce-Mendoza AY, Montes-Zapata EI, Calderón-Meléndez RC, Montelongo-RodrÍguez MJ. Simplified neutrophil isolation protocol. Int J Immunol Immunother. 2020:7(1):041. 10.23937/2378-3672/1410041

[24] Standish AJ , Weiser JN. Human neutrophils kill Streptococcus pneumoniae via serine proteases. J Immunol. 2009:183(4):2602–2609. 10.4049/jimmunol.0900688 19620298

[25] Dahlgren C , Karlsson A. Respiratory burst in human neutrophils. J Immunol Methods. 1999:232(1-2):3–14. 10.1016/S0022-1759(99)00146-5 10618505

[26] Xavier MN , Winter MG, Spees AM, Nguyen K, Atluri VL, Silva TMA, Bäumler AJ, Müller W, Santos RL, Tsolis RM. CD4+ T cell-derived IL-10 promotes Brucella abortus persistence via modulation of macrophage function. PLoS Pathog. 2013:9(6):e1003454. 10.1371/journal.ppat.1003454 23818855 PMC3688575

[27] Liu X , Alli R, Steeves M, Nguyen P, Vogel P, Geiger TL. The T cell response to IL-10 alters cellular dynamics and paradoxically promotes central nervous system autoimmunity. J Immunol. 2012:189(2):669–678. 10.4049/jimmunol.1200607 22711892 PMC3392541

[28] Siwapornchai N , Lee JN, Tchalla EYI, Bhalla M, Yeoh JH, Roggensack SE, Leong JM, Ghanem ENB. Extracellular adenosine enhances the ability of PMNs to kill Streptococcus pneumoniae by inhibiting IL-10 production. J Leukoc Biol. 2020:108(3):867–882. 10.1002/JLB.4MA0120-115RR 32017200 PMC8314384

[29] Bhattacharyya A , Herta T, Conrad C, Frey D, García P, Suttorp N, Hippenstiel S, Zahlten J. Induction of krüppel-like factor 4 mediates polymorphonuclear neutrophil activation in Streptococcus pneumoniae infection. Front Microbiol. 2021:11:582070. 10.3389/fmicb.2020.582070 33613460 PMC7887292

[30] Herring SE , Mao S, Bhalla M, Tchalla EYI, Kramer JM, Ghanem ENB. Mitochondrial ROS production by neutrophils is required for host antimicrobial function against Streptococcus pneumoniae and is controlled by A2B adenosine receptor signaling. PLoS Pathog. 2022:18(11):e1010700. 10.1371/journal.ppat.1010700 36374941 PMC9704767

[31] Hutchins AP , Takahashi Y, Miranda-Saavedra D. Genomic analysis of LPS-stimulated myeloid cells identifies a common pro-inflammatory response but divergent IL-10 anti-inflammatory responses. Sci Rep. 2015:5(1):9100. 10.1038/srep09100 25765318 PMC4650320

[32] Dang PM-C , Elbim C, Marie J-C, Chiandotto M, Gougerot-Pocidalo M-A, El-Benna J, Dang PM-C, Elbim C, Marie J-C, Chiandotto M, et al Anti-inflammatory effect of interleukin-10 on human neutrophil respiratory burst involves inhibition of GM-CSF-induced p47PHOX phosphorylation through a decrease in ERK1/2 activity. FASEB J. 2006:20(9):1504–1506. 10.1096/fj.05-5395fje 16720733

[33] Tamassia N , Calzetti F, Menestrina N, Rossato M, Bazzoni F, Gottin L, Cassatella MA. Circulating neutrophils of septic patients constitutively express IL-10R1 and are promptly responsive to IL-10. Int Immunol. 2008:20(4):535–541. 10.1093/intimm/dxn015 18308712

[34] Benarafa C . Regulation of neutrophil serine proteases by intracellular serpins. In: Geiger M, Wahlmüller F, Furtmüller M, editors. The serpin family: proteins with multiple functions in health and disease. New York (NY): Springer; 2015. p. 59–76. 10.1007/978-3-319-22711-5_5

[35] Elbim C , Reglier H, Fay M, Delarche C, Andrieu V, Benna JE, Gougerot-Pocidalo M-A. Intracellular pool of IL-10 receptors in specific granules of human neutrophils: differential mobilization by proinflammatory mediators. J Immunol. 2001:166(8):5201–5207. 10.4049/jimmunol.166.8.5201 11290804

[36] Roilides E , Katsifa H, Tsaparidou S, Stergiopoulou T, Panteliadis C, Walsh TJ. Interleukin 10 suppresses phagocytic and antihyphal activities of human neutrophils. Cytokine. 2000:12(4):379–387. 10.1006/cyto.1999.0567 10805220

[37] Laichalk LL , Danforth JM, Standiford TJ. Interleukin-10 inhibits neutrophil phagocytic and bactericidal activity. FEMS Immunol Med Microbiol. 1996:15(4):181–187. 10.1111/j.1574-695x.1996.tb00084.x 8908479

[38] Matute-Bello G , Downey G, Moore BB, Groshong SD, Matthay MA, Slutsky AS, Kuebler WM. Group ALI in AS. An official American thoracic society workshop report: features and measurements of experimental acute lung injury in animals. Am J Respir Cell Mol Biol. 2011:44(5):725–738. 10.1165/rcmb.2009-0210st 21531958 PMC7328339

[39] Lim S , Caramori G, Tomita K, Jazrawi E, Oates T, Chung KF, Barnes PJ, Adcock IM. Differential expression of IL-10 receptor by epithelial cells and alveolar macrophages. Allergy. 2004:59(5):505–514. 10.1111/j.1398-9995.2004.00455.x 15080831

[40] Yang S-C , Tsai Y-F, Pan Y-L, Hwang T-L. Understanding the role of neutrophils in acute respiratory distress syndrome. Biomed J. 2021:44(4):439–446. 10.1016/j.bj.2020.09.001 33087299 PMC7481802

[41] Kantrow SP , Shen Z, Jagneaux T, Zhang P, Nelson S. Neutrophil-mediated lung permeability and host defense proteins. Am J Physiol Lung Cell Mol Physiol. 2009:297(4):L738–L745. 10.1152/ajplung.00045.2009 19648288 PMC2770784

[42] Grunwell JR , Giacalone VD, Stephenson S, Margaroli C, Dobosh BS, Brown MR, Fitzpatrick AM, Tirouvanziam R. Neutrophil dysfunction in the airways of children with acute respiratory failure due to lower respiratory tract viral and bacterial coinfections. Sci Rep. 2019:9(1):2874. 10.1038/s41598-019-39726-w 30814584 PMC6393569

[43] Grunwell JR , Stephenson ST, Mohammad AF, Jones K, Mason C, Opolka C, Fitzpatrick AM. Differential type I interferon response and primary airway neutrophil extracellular trap release in children with acute respiratory distress syndrome. Sci Rep. 2020:10(1):19049. 10.1038/s41598-020-76122-1 33149247 PMC7642368

[44] Polverino E , Rosales-Mayor E, Dale GE, Dembowsky K, Torres A. The role of neutrophil elastase inhibitors in lung diseases. Chest. 2017:152(2):249–262. 10.1016/j.chest.2017.03.056 28442313

[45] Domon H , Terao Y. The role of neutrophils and neutrophil elastase in pneumococcal pneumonia. Front Cell Infect Microbiol. 2021:11:615959. 10.3389/fcimb.2021.615959 33796475 PMC8008068

[46] Herta T , Bhattacharyya A, Rosolowski M, Conrad C, Gurtner C, Gruber AD, Ahnert P, Gutbier B, Frey D, Suttorp N, et al Krueppel-Like factor 4 expression in phagocytes regulates early inflammatory response and disease severity in pneumococcal pneumonia. Front Immunol. 2021:12:726135. 10.3389/fimmu.2021.726135 34589087 PMC8473698

[47] Zhang X , Majlessi L, Deriaud E, Leclerc C, Lo-Man R. Coactivation of syk kinase and MyD88 adaptor protein pathways by Bacteria promotes regulatory properties of neutrophils. Immunity. 2009:31(5):761–771. 10.1016/j.immuni.2009.09.016 19913447

[48] Nagamatsu K , Kuwae A, Konaka T, Nagai S, Yoshida S, Eguchi M, Watanabe M, Mimuro H, Koyasu S, Abe A. Bordetella evades the host immune system by inducing IL-10 through a type III effector, BopN. J Exp Med. 2009:206(13):3073–3088. 10.1084/jem.20090494 20008527 PMC2806459

[49] Kelly AM , Leech JM, Doyle SL, McLoughlin RM. Staphylococcus aureus-induced immunosuppression mediated by IL-10 and IL-27 facilitates nasal colonisation. PLoS Pathog. 2022:18(7):e1010647. 10.1371/journal.ppat.1010647 35776778 PMC9282462

[50] Leech JM , Lacey KA, Mulcahy ME, Medina E, McLoughlin RM. IL-10 Plays opposing roles during Staphylococcus aureus systemic and localized infections. J Immunol. 2017:198(6):2352–2365. 10.4049/jimmunol.1601018 28167629 PMC5337812

[51] Dwivedi V , Gautam S, Headley CA, Piergallini T, Torrelles JB, Turner J. IL-10 Receptor blockade delivered simultaneously with Bacillus calmette–guérin vaccination sustains long-term protection against Mycobacterium tuberculosis infection in mice. J Immunol. 2022:208(6):1406–1416. 10.4049/jimmunol.2100900 35181640 PMC11075079

[52] Bebien M , Hensler ME, Davanture S, Hsu L-C, Karin M, Park JM, Alexopoulou L, Liu GY, Nizet V, Lawrence T. The pore-forming toxin β hemolysin/cytolysin triggers p38 MAPK-dependent IL-10 production in macrophages and inhibits innate immunity. PLoS Pathog. 2012:8(7):e1002812. 10.1371/journal.ppat.1002812 22829768 PMC3400567

[53] Desai TJ , Brownfield DG, Krasnow MA. Alveolar progenitor and stem cells in lung development, renewal and cancer. Nature. 2014:507(7491):190–194. 10.1038/nature12930 24499815 PMC4013278

[54] Malinina A , Dikeman D, Westbrook R, Moats M, Gidner S, Poonyagariyagorn H, Walston J, Neptune ER. IL10 Deficiency promotes alveolar enlargement and lymphoid dysmorphogenesis in the aged murine lung. Aging Cell. 2020:19(4):e13130. 10.1111/acel.13130 32170906 PMC7189990

