# Supplementary material for: Neutrophil responsiveness to IL-10 impairs clearance of Streptococcus pneumoniae from the lungs
Source: J Leukoc Biol. 2023 Jun 29;115(1):4–15. doi: 10.1093/jleuko/qiad070 (PMC10768920; doi:10.1093/jleuko/qiad070)
Supplement: qiad070_Supplementary_Data [file qiad070_supplementary_data.pdf]

SUPPORTING INFORMATION

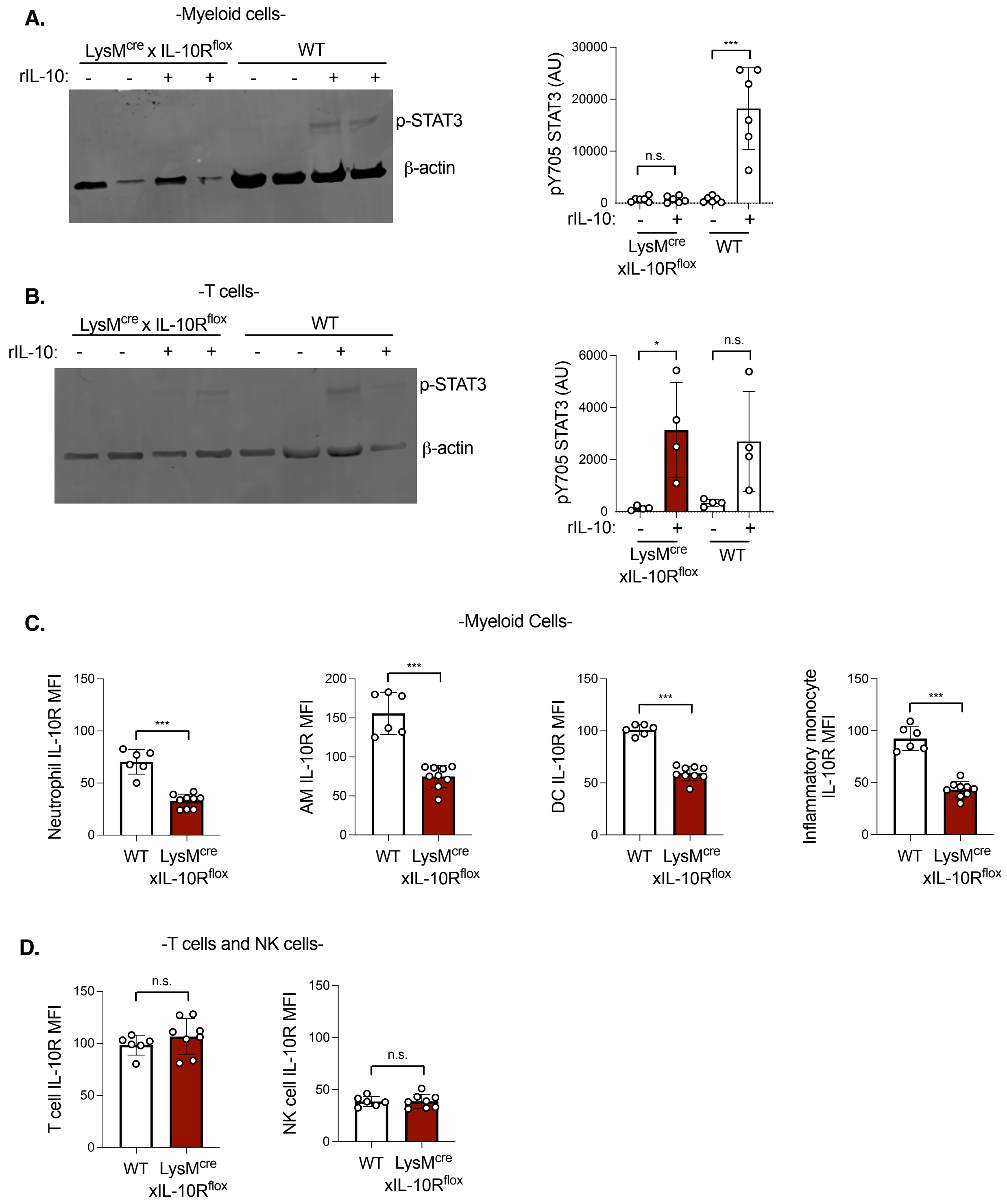

S1 Fig. Abrogation of IL-10R signaling in myeloid cell IL-10R deficient mice. A-B Immunoblot detection of p-STAT3 (Y705) from cell lysates prepared from the spleens of naïve WT or LysM^{cre}xIL-10R^{flox} mice following positive selection for CD64^{+} cells (myeloid cells) (A) or

CD3$^+$ cells (T cells) (B) and +/- 1 h exposure to rIL-10. Densitometry quantification shown as Arbitrary Units (AU) normalized to β-actin loading controls (*n* = cells purified from 4-6 mice/group). **C-D** Median fluorescence intensity (MFI) of IL-10R detected by flow cytometry in the lungs of WT or LysM^{cre}xIL-10R^{flox} mice at 72 h post-infection with *S. pneumoniae* 10^6 CFU/mouse i.t. on neutrophils (CD45$^+$SiglecF$^-$Ly6G$^+$), alveolar macrophages (CD45$^+$SiglecF$^+$CD11b^{low}), dendritic cells (CD45$^+$CD11c$^+$SiglecF$^-$MHCII$^+$, and inflammatory monocytes (CD45$^+$SiglecF$^-$Ly6G$^-$Ly6C$^+$CD11b$^+$) (C) or T cells (CD45$^+$CD3$^+$) and NK cells (CD45$^+$CD3$^-$NK1.1$^+$) (D), *n* = 6-8 mice/group. Data are pooled from three independent experiments and are displayed as mean ± SEM. ***p<.001, *p<.05, two tailed *t*-test.

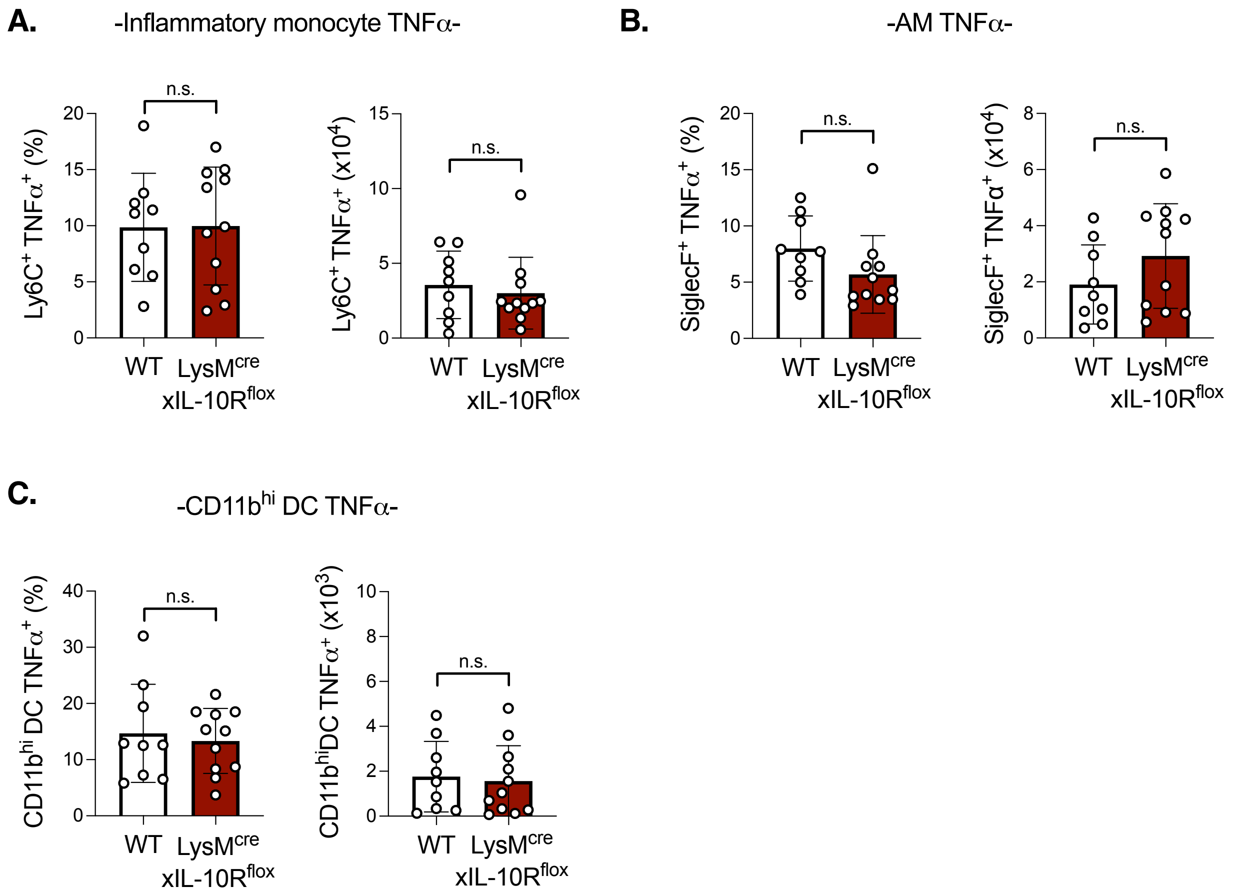

S2 Fig. Myeloid IL-10R deficiency does not impact TNFα expression in AMs, inflammatory monocytes, or CD11b^{hi} DCs. A-C Percentage and total number of TNFα$^+$ inflammatory monocytes (CD45$^+$SiglecF$^-$Ly6G$^-$Ly6C$^+$CD11b$^+$) (A), alveolar macrophages (CD45$^+$SiglecF$^+$CD11b^{low}) (B) and CD11b^{hi} dendritic cells (CD45$^+$CD11c$^+$SiglecF$^-$MHCII$^+$CD11b^{hi}) (C) detected in the lungs by intracellular flow cytometry at 72 h post-infection of WT or LysM^{cre}xIL-10R^{flox} mice with *S. pneumoniae* 10^6 CFU/mouse i.t. (*n* = 10-11 mice/group). Data are pooled from three independent experiments and are displayed as mean ± SEM. *p<.05, two tailed *t*-test.

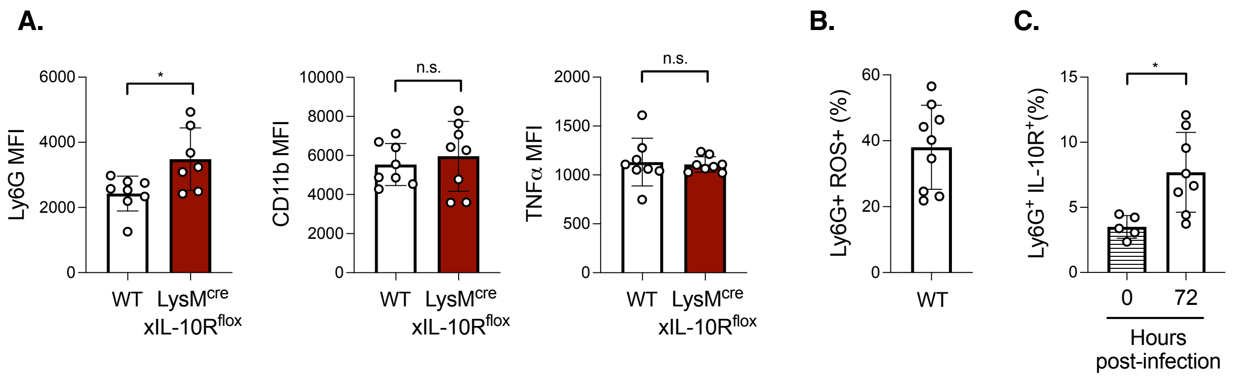

S3 Fig. Impact of myeloid IL-10R deficiency on neutrophils in the lungs of infected mice. **A** MFI for neutrophil Ly6G, CD11b, and TNFα detected in the lungs by intracellular flow cytometry at 72 h post-infection of WT or LysM^{cre}xIL-10R^{flox} mice with *S. pneumoniae* 10^6 CFU/mouse i.t. (*n* = 7-8 mice/group). **B** Percent reactive oxygen species (ROS) expression on neutrophils in the lungs of mice 72 h post-infection with *S. pneumoniae* detected by flow cytometry. **C** Percent IL-10R expression on neutrophils in the lungs of naïve mice or at 72 h post-infection with *S. pneumoniae* detected by flow cytometry. Data are pooled from three independent experiments and are displayed as mean ± SEM. *p<.05, two tailed *t*-test.

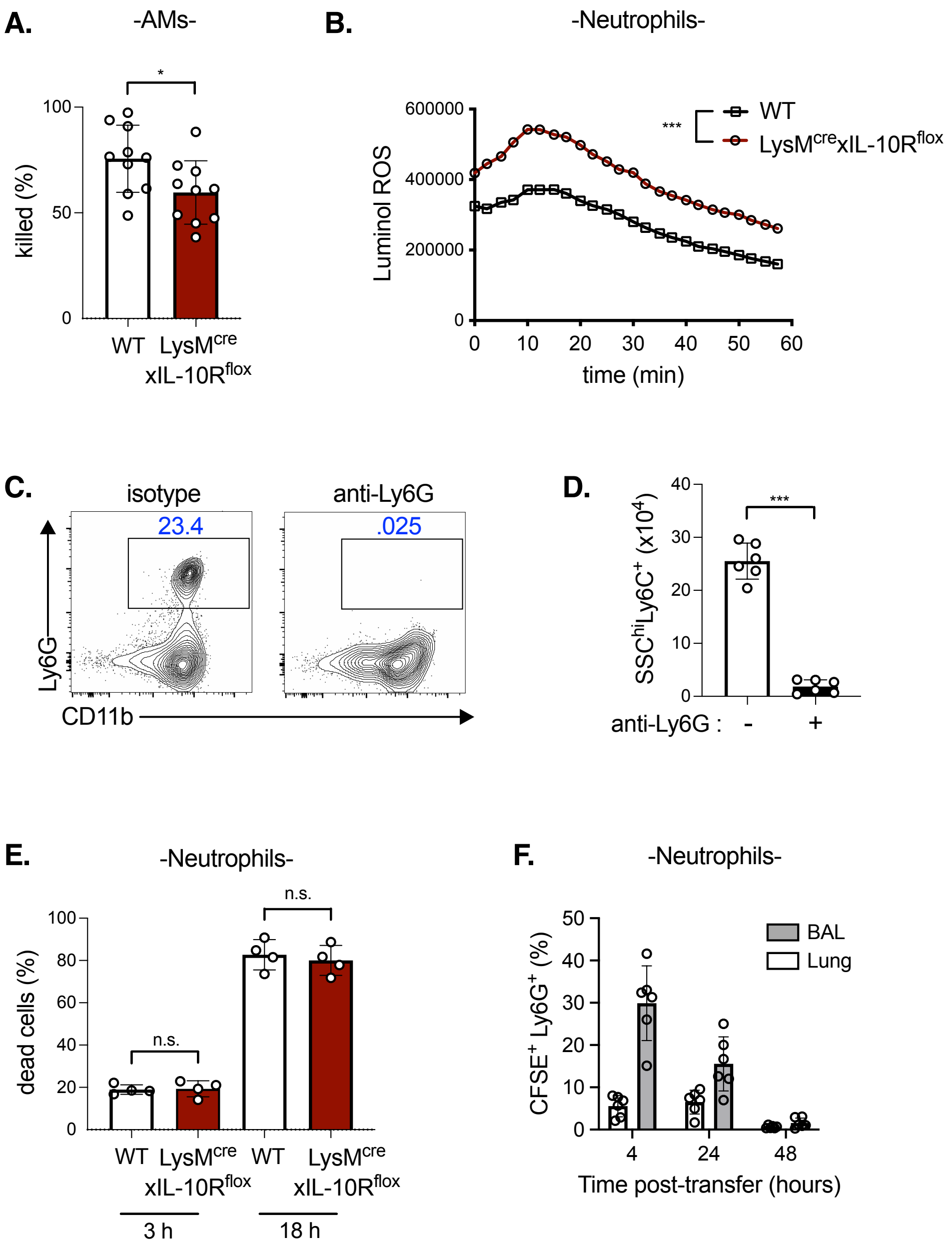

S4 Fig. AM killing of *S. pneumoniae*, neutrophil luminol ROS, and donor neutrophil survival for cells from WT versus myeloid cell IL-10R deficient mice. A Percent of *S. pneumoniae* killed by alveolar macrophages purified from naïve WT or LysM^{cre}xIL-10R^{flox} mice (*n* = cells isolated from 10 mice/group). **B** ROS production over time measured by luminol chemiluminescence. **C-D** Percentage of Ly6G^{+}CD11b^{+} neutrophils (C) and SSC^{hi}Ly6C^{+} neutrophils (D) detected in the lungs of mice treated with isotype control (-) or anti-Ly6G antibodies (+) i.p. 24 h prior to *S. pneumoniae* infection (*n* = 6 mice/group). **E** Percentage of dead cells determined by viability staining on neutrophils purified from the bone marrow of naïve WT or LysM^{cre}xIL-10R^{flox} mice (*n* = cells isolated from 4 mice/group). **F** Percentage of CFSE+ donor neutrophils in the bronchoalveolar lavage (BAL) or lungs of recipient mice infected with *S. pneumoniae* 24 h prior to injection with donor neutrophils detected by flow cytometry (*n* = 6-9 recipient mice/group). Data are pooled from two (E, F) or three (A) independent experiments, or

representative one of three experiments (B-D) and are displayed as mean ± SEM. ***p<.001, *p<.05, two tailed *t*-test.

Table I. Score of histopathologic changes in lungs.

Score	Cell Infiltrate	Hemorrhage	Swelling of Alveolar Septa	Lung Damage	Damage Extension
0	Normal amounts of alveolar macrophages. No intra-alveolar inflammatory cells. No bronchiolitis and no inflammatory cells within lumen.	No intra-alveolar red blood cells and/or fibrin	None	Normal lung architecture	No lesions*
1	Any increase in macrophages or inflammatory cells in alveoli and/or bronchioles	Some intra-alveolar red blood cells and/or fibrin	Some / patchy	Minimal change in lung architecture	Focal or small lesions
2	Moderate to severe increase in macrophages or inflammatory cells in alveoli and/or bronchioles	Most of lung with intra-alveolar red blood cells and/or fibrin	Diffuse, but recognizable alveoli	Moderate change in lung architecture with alveoli and bronchioles distinguishable	Large or diffuse lesions
3	Obliterative bronchiolitis and/or lung architecture obscured by inflammatory cell infiltrates		Diffuse and obscuring lung architecture	No recognizable lung structures	

Scored parameters were evaluated on digitized whole slide images with Aperio ImageScope software. *Lesions were defined as discrete areas of inflammation and/or architectural disruption. Scoring adapted from(1).

1. González LA, Melo-González F, Sebastián VP, Vallejos OP, Noguera LP, Suazo ID, et al. Characterization of the Anti-Inflammatory Capacity of IL-10-Producing Neutrophils in Response to *Streptococcus pneumoniae* Infection. Front Immunol. 2021;12:638917.
